# Transcutaneous bilirubin measurements in preterm infants: the impact of race, age, and phototherapy

**DOI:** 10.1038/s41372-025-02558-w

**Published:** 2026-01-05

**Authors:** Natasha Cordero, Anna Petrova, Amrryn Halari, Thomas Hegyi

**Affiliations:** The Division of Neonatology, Department of Pediatrics, Robert Wood Johnson Medical School, Rutgers University, New Brunswick, NJ USA

**Keywords:** Diagnostic markers, Laboratory techniques and procedures

## Abstract

**Background:**

Monitoring bilirubin in preterm neonates is essential to prevent neurotoxicity. Total serum bilirubin (TSB) remains the diagnostic standard, while transcutaneous bilirubinometry (TcB) offers a rapid, non-invasive, and cost-effective alternative. However, TcB’s accuracy in preterm infants, especially during phototherapy and across racial or gestational groups, remains uncertain.

**Objective:**

To assess concordance between TcB and TSB in preterm neonates across gestational ages, racial/ethnic groups, and phototherapy exposure.

**Methods:**

We analyzed 330 paired TcB-TSB readings from 113 preterm infants (postnatal age 5–408 h, mean ± SD: 91.8 ± 64.2) in a NICU. Infants were categorized by gestational age (<28, 28–32, 32–36 weeks), including self-reported maternal race and ethnicity. We employed correlation analysis, paired t-tests, linear regression, and Bland-Altman plots. TcB was evaluated under phototherapy (exposed vs. patched skin) in relation to escalation of care thresholds (ECT).

**Results:**

TcB and TSB were strongly correlated overall (r = 0.822), but the level of agreement varied by gestational age and phototherapy exposure. Accuracy declined with phototherapy, especially on exposed skin. Bland-Altman analysis showed that TSB and TcB are not interchangeable. Finally, TcB-ECT concordance was low.

**Conclusion:**

TcB correlates with TSB but lacks sufficient accuracy for standalone use in preterm infants, particularly during phototherapy. Caution is called for in clinical application.

## Introduction

Neonatal hyperbilirubinemia affects over 80% of newborns, and when severe, may lead to irreversible neurological injury, including kernicterus spectrum disorder. Preterm infants are particularly vulnerable because of immature hepatic conjugation, underdeveloped blood-brain barrier function, and reduced capacity for bilirubin binding and clearance. Prompt recognition and accurate monitoring of bilirubin levels are essential to prevent neurotoxicity in this high-risk population. Total serum bilirubin (TSB) remains the gold standard for assessing the risk of hyperbilirubinemia. However, it requires invasive blood sampling, which can be painful and may contribute to iatrogenic anemia, particularly in very preterm infants.

To reduce the burden of repeated blood draws, transcutaneous bilirubin (TcB) devices were introduced in 1980 by Yamanouchi [1]. These non-invasive devices estimate bilirubin through skin light reflectance, providing rapid bedside measurements. In term and late preterm infants, TcB generally correlates well with TSB [2–5], and the American Academy of Pediatrics (AAP) recommends TcB use for infants ≥35 weeks’ gestation as a screening tool [5].

Despite its advantages, the role of TcB in preterm infants remains controversial [6–11]. Prior studies indicate that TcB may underestimate TSB during phototherapy, particularly on exposed skin, limiting its reliability for monitoring treatment response [12]. Furthermore, TcB accuracy is influenced by multiple factors, including gestational age, skin pigmentation, body weight, and environmental exposures. Existing guidelines reflect this uncertainty: the National Institute for Health and Care Excellence (NICE) in the United Kingdom recommends against TcB use in infants <35 weeks’ gestation (NICE CG98), and the AAP advises caution in preterm infants when making therapeutic decisions.

Several additional variables can impact the relationship between TcB and TSB, particularly in preterm infants, including skin thickness, melanin content, hemoglobin concentration, serum albumin, postnatal dermal maturation, hydration status, and exposure to environmental light. Genetic and racial factors may further modify skin reflectance; however, prior studies have often relied on maternal self-reported race and ethnicity, overlooking the paternal contribution. These factors complicate the interpretation of TcB measurements and underscore the importance of careful validation in specific populations.

Most published studies in preterm neonates have been limited by small sample sizes, broad gestational groupings, and inadequate representation of very preterm infants. Moreover, few investigations provide quantitative data on TcB performance stratified by gestational age, phototherapy exposure, or racial/ethnic subgroups, and confounding variables such as skin pigmentation and serum albumin are seldom reported.

To address these gaps, we conducted a prospective observational study of preterm infants admitted to our NICU, categorized by relevant gestational age groups: extremely preterm (<28 weeks), very preterm (28–31 weeks), and late preterm (32–36 weeks). We collected paired TcB and TSB measurements before and during phototherapy and recorded maternal race/ethnicity along with key clinical variables such as birth weight and postnatal age. Our primary goals were to determine the agreement between TcB and TSB across different gestational groups, examine the effects of phototherapy exposure, and explore potential differences based on maternal racial/ethnic background. Secondary goals included assessing how birth weight and postnatal age may influence the accuracy of TcB. This study aims to clarify the circumstances under which TcB can serve as a reliable screening method, while emphasizing that TSB remains the standard for therapeutic decisions in preterm infants.

## Methods

This prospective observational study, approved for human research by the Rutgers University Institutional Review Board, was conducted in the neonatal intensive care unit (NICU) of Bristol-Myers Squibb Children’s Hospital at Robert Wood Johnson University Hospital from January 8, 2023, to April 8, 2025. Based on preliminary estimates for a pilot evaluation, a target sample size of approximately 300 paired TcB and TSB measurements was established.

### Study population

Preterm infants born before 36 weeks of gestation who survived more than 12 h were eligible for enrollment. Informed consent was obtained from a parent or legal guardian, and approval from the attending physician was needed. Infants with significant congenital anomalies or chromosomal abnormalities were excluded to minimize confounding factors. Maternal race and ethnicity were self-reported; race was categorized as Black, White, or Asian infants, while ethnicity was classified as Hispanic or non-Hispanic individuals. Gestational age was determined using the most accurate obstetric estimate available.

### Bilirubin measurements

TSB was measured using the standard diazo method in the hospital clinical laboratory. TcB measurements were obtained within 60 min of TSB collection using the BiliCheck device (Philips, The Netherlands), a non-invasive spectrophotometric instrument. Each TcB value corresponded to the sternal skin site. Infants were grouped by gestational age into three categories: <28 weeks, 28–32 weeks, and >32 weeks.

### Escalation-of-Care Threshold (ECT) analysis

To evaluate clinical equivalence between TcB and TSB, we utilized the escalation-of-care threshold (ECT) introduced in the 2022 American Academy of Pediatrics (AAP) guidelines for newborns 35 weeks or higher and adopted it to our study [5]. The ECT threshold acts as a clinical signal for increased monitoring and potential intervention before reaching the treatment level [13] and was defined as the phototherapy treatment threshold (PTT) minus 3 mg/dL. For each TSB and TcB value, we calculated the difference from the age-specific PTT, creating a continuous measure of proximity to treatment initiation. This enabled a comparative evaluation of TcB and TSB performance in clinical decision-making.

### Phototherapy protocol and skin bleaching substudy

Phototherapy was administered using eight neoBLUE LED lamps (Natus Medical Inc., Middleton, WI), positioned above the infant in an incubator. The lamps delivered an irradiance of 30–40 µW/cm²/nm, as measured by a calibrated photometer. The distance of the lamps and exposure conditions were standardized. In a subgroup of infants receiving phototherapy, TcB was measured from two skin sites at 60-minute intervals over 4 h: (1) a fully unpatched sternal area (TcBUP) and (2) an adjacent area protected by a 2.5 cm opaque patch (TcBP). This setup enabled the assessment of phototherapy-induced skin bleaching and its impact on TcB reliability.

### Statistical analysis

Spearman’s rank correlation was applied to assess the associations among TcB, TSB, gestational age, and postnatal age. Bland-Altman plots were generated to evaluate the agreement between TcB and TSB, calculating the mean bias and 95% limits of agreement (mean ± 1.96 SD). For each TcB-TSB pair, the absolute difference and average value were computed. Fisher’s r-to-z transformation was employed to analyze the differences in TcB-TSB correlation by race. This transformation is a statistical method used to normalize correlation coefficients, since the distribution of *r* (bounded between –1 and +1) is not normally distributed, especially at extreme values. Paired t-tests were conducted to compare the mean TcB and TSB values, and ANOVA was used to assess differences across gestational age groups. Stepwise linear regression identified independent predictors of TcB-TSB discrepancy. Statistical significance was defined as a two-sided p ≤ 0.05. Results are presented as means, standard deviations, correlation coefficients (r), regression coefficients (β), and 95% confidence intervals (CI).

In the phototherapy subgroup, changes in TcBUP and TcBP were analyzed over time to quantify bleaching effects. TcBUP-to-TcBP ratios were calculated at each interval, and both hourly and cumulative bleaching rates were reported. Additionally, the effects of GA, BW, and postnatal age on the differences in TcBP and TcBUP were examined.

## Results

A total of 113 preterm infants were enrolled, with a mean birth weight of 1862 ± 656 grams and a gestational age of 32.4 ± 2.9 weeks. The racial and ethnic distribution included 11 Asian, 21 Black, 34 Hispanic, 43 White, and four multiracial infants. By gestational age (GA), 12 infants were in Group 1 (<28 weeks), 29 were in Group 2 (28–32 weeks), and 72 were in Group 3 (>32 weeks). Forty-four infants received phototherapy during the study, while 69 did not.

### TcB and TSB correlation

**Fig. 1:**
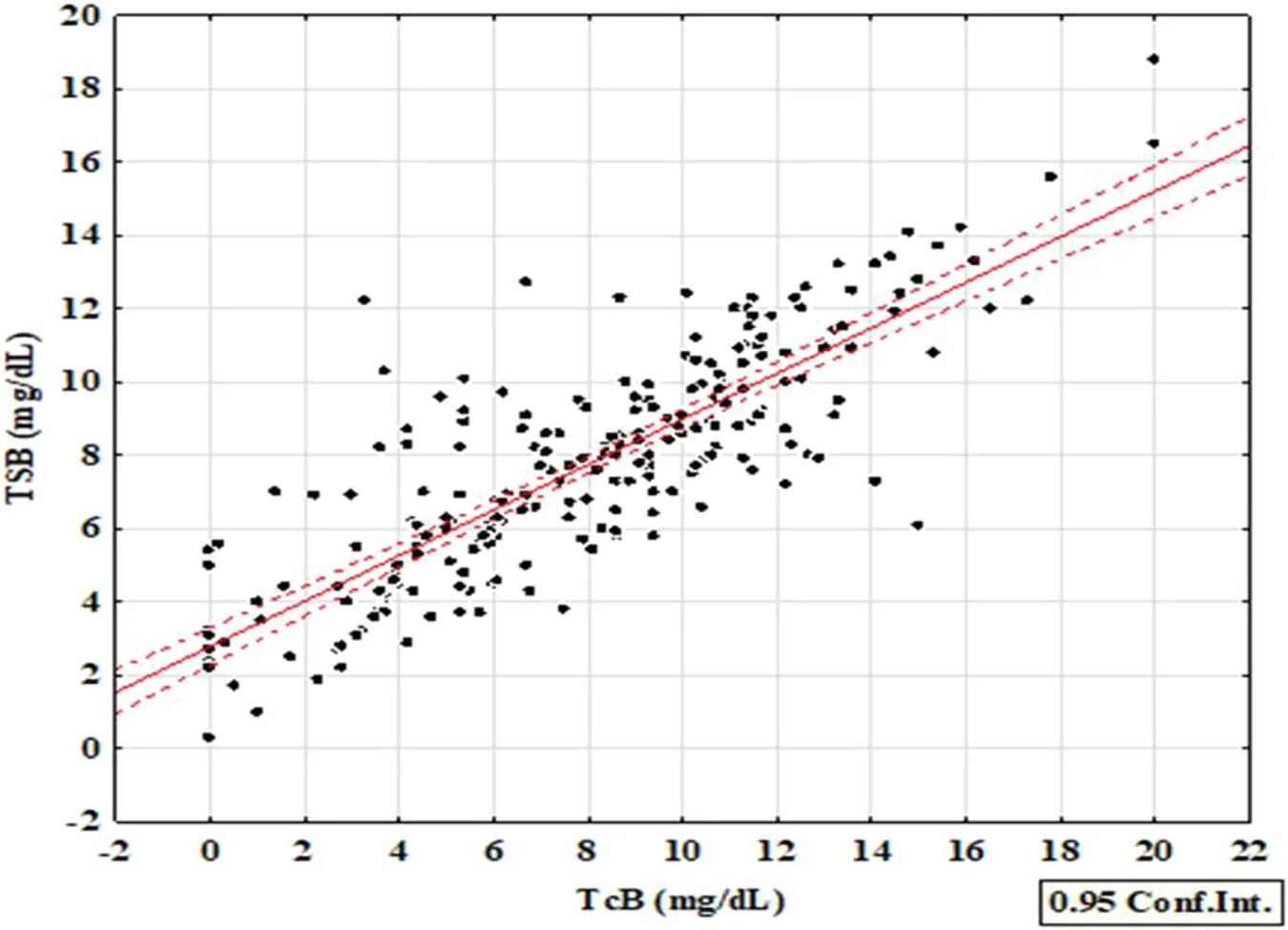
Correlation between TSB and TcB in the Study Population. TSB = 2.7880 + 0.62100*TcB, Correlation r = 0.822.

**Fig. 2:**
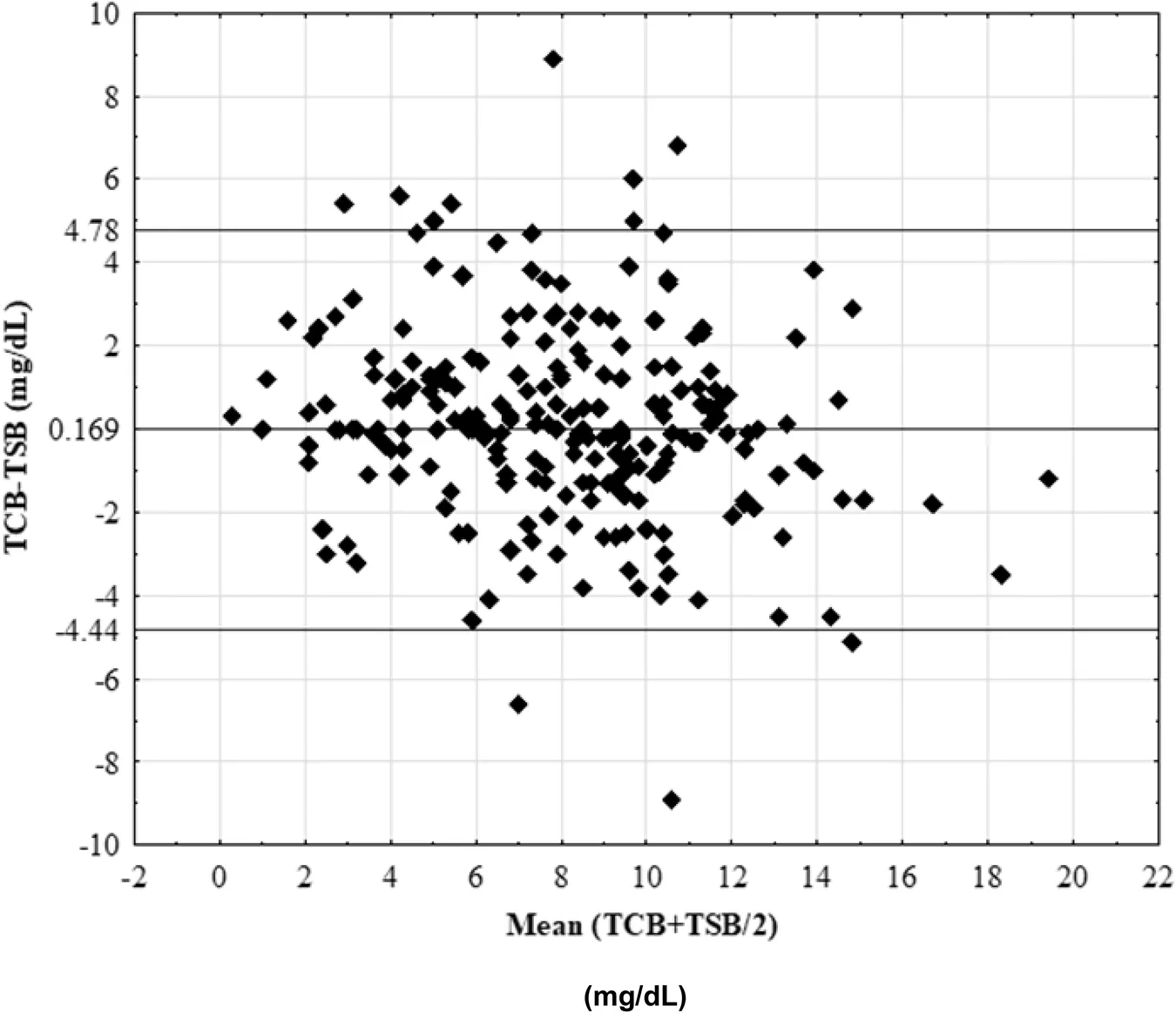
Bland-Altman Limits of Agreement Plot.

We collected 330 paired TSB and TcB samples at a mean postnatal age of 91.8 ± 64.2 h. Of these, 44 were gathered during phototherapy, while 286 were collected without treatment. TSB and TcB values showed a positive correlation with GA (r = 0.389 and r = 0.329, respectively; p < 0.01). Testing age was weakly associated with TSB (r = 0.111, p < 0.05) but not with TcB (r = 0.031, NS). Overall, TSB and TcB were strongly correlated (r = 0.822; R² = 0.676, Fig. 1). The Bland-Altman plot (Fig. 2) indicated a mean TSB-TcB difference of 0.17 mg/dl, suggesting that TcB readings slightly overestimate TSB on average. However, a broad range of agreement shows substantial variability between the two methods, with a significant number of comparisons falling outside the ± 2 mg/dl range, indicating inconsistencies at both low and high bilirubin levels. Of concern is the presence of heteroscedasticity, meaning the error increases with higher bilirubin levels. In untreated infants, the correlation was stronger (r = 0.862; R² = 0.743) than in those undergoing phototherapy (r = 0.749; R² = 0.561). Racial differences had minimal impact on the TcB-TSB correlation. Correlations ranged from r = 0.725 in Asian infants to r = 0.800 in White infants. Off phototherapy, Hispanic infants showed the strongest correlation (r = 0.863) and Black infants the weakest (r = 0.833). Under phototherapy, correlations varied widely—from r = 0.352 in Hispanic infants to r = 0.949 in Black infants—likely due to small sample sizes.

### Gestational age subgroup analysis

Table 1 summarizes gestational age (GA), birth weight, and postnatal age at testing for each GA-based group. We recognized that the number of infants in Group I was limited, which may impact the results. Infants in Group 1 exhibited lower TcB and TSB levels, higher usage of phototherapy, and lower breastfeeding rates. The correlation between TcB and TSB remained significant within each GA group (Group 1: r = 0.862; Group 2: r = 0.752; Group 3: r = 0.797). Regression analysis indicated a positive association between the TcB–TSB difference and GA (β = 0.144, 95% CI: 0.006–0.283; p < 0.01), as well as a negative association with the average bilirubin level (TSB+TcB/2; β = –0.239, 95% CI: –0.379 to –0.101; p < 0.001). The TcB-TSB correlation was consistently stronger in untreated infants across all GA groups.

**Table 1:** Demographic and outcome data of gestational age groups.

	Group 1	Group 2	Group 3
GA (weeks)^a^	26.1 (25.6, 26.5)	30.6 (30.2, 30.8)	34.3 (34.2, 34.4)**
BW (grams)^a^	894 (779, 1099)	1449 (1363, 1534)	2173 (2088, 2257)**
Testing age (hours)^a^	99.3 (68.3, 130.2)	100.1 (84.5, 115.6)	80.8 (71.4, 90.2)
TcB/TSB samples, n	27	119	174
TcB/TSB samples under phototherapy, n	10	23	11
TcB, (mg/dL)^a^	3.7 (2.7, 4.8)	8.0 (7.1, 8.9)	8.9 (8.2, 9.6)
TSB, (mg/dL)^a^	4.3 (3.7, 5.0)*	7.7 (7.1, 8.3)	8.5 (8.0, 9.1)
TcB-TSB, (mg/dL)^a^	–0.58 (–1.78, 0.62)*	0.30 (−0.77, 1.37)	0.40 (–0.50, 1.25)
TcB +TSB/2^a^	4.3 (3.5, 5.1)**	8.0 (7.3, 8.6)	8.8 (8.2, 9.4)
TcB/TSB correlation (r)	0.862	0.752	0.797

^a^median, 25th, and 75th percentiles

*p < 0.05 **p < 0.01

### Escalation-of-Care threshold (ECT) concordance

Among 326 TSB-TcB pairs, the mean difference between ECT-TSB and ECT-TcB was 0.3 mg/dL. TSB identified 59 samples below the ECT threshold (i.e., candidates for phototherapy). Of these, TcB values identified 21 as treatment candidates, 22 were already receiving treatment, and 18 did not meet the treatment criteria, indicating limited agreement in some cases.

### Phototherapy group characteristics

Fourteen infants were analyzed during phototherapy: 78.6% were female, 42.8% Hispanic infants, and 28.6% were White infants. Their GA ranged from 28 to 35 weeks (mean 30.8; 95% CI: 28.8–32.9), and birth weights varied from 734 to 3230 g (mean 1807; 95% CI: 1596–2017). Bilirubin samples were collected between 11 and 297 h postnatal (mean 96 h; 95% CI: 79–114). A strong correlation between TSB and TcB was observed (r = 0.75, p < 0.001), and paired t-testing showed no significant difference in means (TSB: 8.9 ± 3.2 mg/dL; TcB: 8.4 ± 4.4 mg/dL; p = 0.36; mean difference: 0.405 mg/dL, 95% CI: –0.482 to 1.29). However, Bland-Altman analysis revealed wide limits of agreement, indicating inadequate interchangeability. Multivariable regression found no significant associations between absolute TSB–TcB differences and gestational age, birth weight, or age at testing.

### Skin exposure and bleaching analysis

Seventy TcB sample pairs were collected from phototherapy-exposed (TcBUP) and protected (TcBP) skin at baseline and every hour for 4 h. Significant differences appeared after 1 h, with TcBUP levels progressively declining (p < 0.01). The TcBUP/TcBP ratio decreased from 0.91 ± 0.86 at baseline to 0.44 ± 0.23 by hour 4. A paired comparison at each hour showed a mean reduction of TcBUP compared to TcBP of 4.1 ± 2.3 at h 1, 5.6 ± 2.3 at h 2, 5.4 ± 2.3 at h 3, and 5.1 ± 2.1 at h 4. Absolute changes in TcBUP from baseline (h 0) at h 1–4 were 4.4 ± 3.0, 6.3 ± 2.6, 7.0 ± 2.8, and 7.4 ± 0.7 mg/dL, respectively.

Regression analysis (Table 2) revealed that the absolute difference (AD) between TcBP and TcBUP increased with gestational age after 1–2 h of phototherapy and with birth weight after 1 and 4 h of phototherapy. A significant inverse relationship between AD and age at testing was observed after 2 h of therapy. TcBP levels remained relatively stable during the first 2 h, with reductions noted at hours 3 and 4, indicating delayed phototherapy effects on protected skin.

**Table 2:** Prediction of absolute difference of TcBs by GA, BW, and Age (β, 95%CI).

Timeline	GA (weeks)	BW (grams)	Age (hours)
1 h	1.492 (1.099, 1.884)***	1.492 (–1.885, –1.102)***	–0.38 (–0.338, 0.061)
2 h	0.942 (0.525, 1.359)***	–0.326 (–0.743, 0.090)	–0.218 (–0.431, –0.006)*
3 h	0.257 (–0.337, 0.852)	0.045 (–0.549, 0.640)	–0.239 (–0.537, 0.057)
4 h	–0.939 (–1.427, –0.451)**	1.155 (0.668, 1.642)***	–0.229 (–0.479, 0.018)

*P < 0.05; **P < 0.01; ***P < 0.0001

## Discussion

Our study investigated the relationship between TcB and TSB in preterm infants, with a particular focus on the impact of phototherapy. These findings contribute to the growing understanding of TcB’s utility in this high-risk population, where hyperbilirubinemia is common, potentially severe, and best managed through early detection. While TSB remains the diagnostic gold standard, its invasive nature, resulting in blood loss and procedural pain, poses risks for preterm neonates [14]. Integrating noninvasive TcB measurements into routine care, as successfully implemented in more mature infants [5], remains a priority in neonatal practice.

Despite its established role in term and late-preterm infants [13], the adoption of TcB in neonates <35 weeks’ gestation remains controversial [9, 15]. Multiple studies report that TcB underestimates TSB at higher concentrations and during phototherapy, potentially delaying treatment [16, 17]. Factors such as skin thickness, pigmentation, and epidermal and dermal immaturity affect light scattering and absorption, thereby reducing the reliability of TcB in very low birth weight infants [8]. Consequently, extreme caution has been recommended for extremely preterm neonates; Cucuy [17] and Murli [18] observed poor TcB-TSB correlation regardless of phototherapy exposure. Nevertheless, under controlled conditions with device calibration and consistent protocols, TcB can achieve acceptable correlation with TSB [2].

The variability across studies likely reflects methodological differences, including study design, device selection, measurement site, and underlying skin biology. Thinner, immature skin alters bilirubin’s optical properties through variations in light scattering, absorption, hydration, and capillary perfusion [3], contributing to hesitancy about universal TcB adoption in NICUs, where confirmatory serum testing remains necessary in many cases.

In our cohort, TcB and TSB levels were significantly correlated across all infants, with correlations improving when samples obtained during phototherapy were excluded [19]. Gestational age and birth weight had minimal impact, as each subgroup demonstrated significant correlations between TcB and TSB. Postnatal age showed only a weak association with TSB and no association with TcB.

Race appeared to have minimal impact on TcB accuracy in our study. Among the four racial groups analyzed, we observed no substantial influence on the TcB-TSB relationship. While earlier reports suggested that darker skin pigmentation could lead to TcB overestimating TSB, our findings align with more recent studies indicating that devices such as BiliCheck mitigate these effects in preterm populations [20, 21]. Nonetheless, Engle noted that TcB tended to underestimate TSB at higher bilirubin levels in predominantly Hispanic infants [16], highlighting the necessity for contextual interpretation of results. The limited number of phototherapy-exposed samples in our cohort restricts the generalizability of these conclusions.

Although nomograms have been created using TcB data, few studies have focused on preterm infants. Kaplan’s analysis of 19 TcB nomograms across diverse populations revealed substantial variability, most likely attributable to underlying racial and ethnic differences [22].

In a distinct aspect of our analysis, we evaluated how TcB and TSB predicted the need for phototherapy using ECT as a guide. TSB-based criteria resulted in significantly more recommendations for phototherapy than TcB, with a treatment ratio of 2.8:1 (Chi-square 20.57, p < 0.05). This discrepancy has important clinical implications, particularly considering recent concerns over the overuse of phototherapy: more than 1000 infants may receive phototherapy to prevent a single case of extreme hyperbilirubinemia requiring exchange transfusion [23]. This is further complicated by many clinicians who start phototherapy treatment at lower levels than those recommended by the AAP [24].

Phototherapy universally weakened the TcB-TSB relationship across subgroups, including variations by gestational age, postnatal age, and race. Although statistically significant correlations remained, the agreement between TcB and TSB during phototherapy was consistently poorer, aligning with our findings and those of prior investigations [19]. The meta-analysis from Ten Kate et al. and the systematic review by Hynes et al. Ten Kate et al. showed that TcB measurements are generally accurate before phototherapy, with a pooled mean difference (MD) of 2.5 μmol/L and relatively narrow limits of agreement, supporting TcB as a reliable screening tool prior to treatment. However, during and after phototherapy, the accuracy of TcB was highly variable and site dependent. TcB measured on covered skin during phototherapy showed good agreement (MD –0.3 μmol/L), but on uncovered skin, TcB significantly underestimated TSB (MD –28.6 μmol/L). After phototherapy, both covered and uncovered skin sites showed substantial underestimation (MD –34.3 μmol/L and –21.1  μmol/L, respectively), with wide limits of agreement, which raises concerns about clinical reliability in these contexts. The best agreement was found at the forehead, with no significant difference observed between term and preterm neonates. Therefore, TcB levels measured on exposed skin are unreliable under phototherapy, likely due to the biological and optical changes induced by light exposure, which may lead to inaccurate TSB levels and necessitate confirmatory blood testing for safe management.

Phototherapy works by converting bilirubin into water-soluble isomers, which facilitates excretion and reduces dermal bilirubin stores [2]. These manifest clinically as a visible lightening of the skin, especially in exposed areas, corresponding to decreased TcB values. However, we and others have observed that this “bleaching effect” diminishes with prolonged exposure, even when the light intensity remains constant. In our earlier study, TcB values in preterm infants declined sharply during the initial hours of white light phototherapy, then plateaued at –3.4, –1.6, +0.3, and +0.4 units/hour over the first to 4 h, respectively [25, 26].

The tapering of TcB decline may reflect a redistribution of bilirubin from the skin into circulation, where light-induced photoisomers are mobilized, reducing the dermal bilirubin available for detection [22]. This process decreases the skin’s yellow pigmentation and limits further TcB decline, even with continued therapy. As shown in our prior work, the rate of bilirubin clearance is modulated by both the wavelength and intensity of the phototherapy light [25]. The diminishing TcB response may also explain the efficacy of intermittent phototherapy, which allows dermal bilirubin to reaccumulate during off-light periods, restoring photo-conversion efficiency when therapy resumes [27, 28].

Our findings support the use of patched skin sites for TcB measurement during phototherapy. TcB values obtained from shielded areas remained more closely aligned with TSB levels, in contrast to the increasing discrepancy seen with exposed sites. The TcBUP/TcBP ratio decreased from 0.91 to 0.44 over a 4 h period of light exposure, indicating a progressive loss of reliability at exposed sites. Stepwise regression revealed that the size of this difference was influenced by gestational age, birth weight, postnatal age, and duration of exposure. These results are consistent with studies showing that patched site readings correlate better with serum bilirubin and are more clinically dependable [16, 29]. To enhance consistency, Maisels recommended standardized patching and measurement protocols for clinical practice [30].

## Conclusion

Just as in term infants, TcB measurement in the preterm population should be limited to a screening role, but TSB should be used for therapeutic decisions. In premature infants, while TcB correlates reasonably well with TSB on average, our analysis reveals wide limits of agreement and increased variability at higher bilirubin levels, emphasizing the need for caution when using TcB alone in clinical decision-making, particularly at elevated bilirubin levels. Therefore, despite the strong overall correlation, the clinical utility of TcB is limited by the degree of imprecision, as observed in prior studies. At lower bilirubin levels, TcB may underestimate actual serum values, potentially delaying necessary confirmatory testing and timely initiation of therapy. At higher levels, TcB may overestimate risk, leading to unnecessary blood draws, increased parental anxiety, and unwarranted use of healthcare resources, particularly phototherapy. These limitations highlight the importance of interpreting TcB results within the broader clinical context and reinforce the need for confirmatory TSB measurements when TcB values approach treatment thresholds. Another limitation of our study is the reliance on maternal self-reported race and ethnicity for participant categorization. While maternal reporting is a common practice in neonatal research, it may not fully capture the infant’s genetic ancestry, particularly in cases where parents are of different racial or ethnic backgrounds. Consequently, some infants may be misclassified or not accurately represented by the maternal category, potentially obscuring associations between race/ethnicity and clinical outcomes. Future studies could benefit from collecting more granular data on both parents’ race and ethnicity to examine the role of these factors more accurately in neonatal outcomes.

## Summary

What is Known-What is New

### What is Known

- Monitoring bilirubin levels in preterm infants is critical to prevent bilirubin-induced neurotoxicity. Total serum bilirubin (TSB) remains the gold standard for diagnosis and management; however, transcutaneous bilirubinometry (TcB) offers a rapid, non-invasive, and cost-effective screening alternative. Prior studies have demonstrated strong correlations between TcB and TSB in term infants, but accuracy is reduced in preterm populations—especially during phototherapy or in infants with darker skin pigmentation—leading to uncertainty regarding its clinical reliability in these groups.

### What is New

- This study confirms a strong overall correlation between TcB and TSB in preterm neonates but identifies significant variability by gestational age and phototherapy exposure. TcB accuracy declined notably on phototherapy-exposed skin, and Bland–Altman analysis demonstrated that TcB and TSB are not interchangeable. Furthermore, concordance between TcB and escalation-of-care thresholds was poor, indicating potential risk if TcB is used alone for decision-making. These findings underscore the importance of continued reliance on serum measurements and cautious interpretation of TcB values in preterm infants, particularly during and after phototherapy.

## Data Availability

The data that support the findings of this study are not openly available due to reasons of sensitivity and are available from the corresponding author upon reasonable request.
